# Gut Metabolite Indoleacrylic Acid Suppresses Osteoclast Formation by AHR mediated NF-κB Signaling Pathway

**DOI:** 10.7150/ijbs.124766

**Published:** 2026-01-01

**Authors:** Jinwu Bai, Gao Si, Ruideng Wang, Shilong Su, Jixing Fan, Xi He, Yang Lv, Shan Gao, Fang Zhou

**Affiliations:** 1 Department of Orthopedics, Peking University Third Hospital, Beijing 100191, China.; 2 Engineering Research Center of Bone and Joint Precision Medicine, Peking University Third Hospital, Beijing 100191, China.; 3 Department of Trauma Orthopedics, Shenzhen Second People's Hospital (The First Affiliated Hospital of Shenzhen University), Shenzhen, 518000, China.; 4 School of Biological Sciences and Medical Engineering, Beihang University, Beijing 100191, China.

**Keywords:** indoleacrylic acid, osteoclast formation, gut microbiota, aryl hydrocarbon receptor, NF-κB signaling pathway

## Abstract

Bone homeostasis relies on the coordinated activities of bone-forming osteoblasts and bone-resorbing osteoclasts. Disruption of this balance leads to osteoporosis, a highly prevalent bone disease with substantial health impacts in middle-aged and older adults. There is accumulating evidence linking the development of osteoporosis to alterations in the gut microbiota and its metabolite profile. The gut metabolite indole and its derivatives were shown to have beneficial effects in multiple metabolic diseases. However, their effects on bone homeostasis remain unclear. This study identified alterations in the gut microbiota and decreases in levels of tryptophan metabolites in an ovariectomized (OVX) estrogen deficiency-induced osteoporosis mouse model, characterized by decreased abundance of *Lactobacillus* and *Clostridium* species in the gut and reduced serum levels of indoleacrylic acid (IA), indoleacetic acid (IAA), and indolepropionic acid (IPA). IA showed a significant positive correlation with bone mass. Specifically, IA inhibited RANKL-induced aryl hydrocarbon receptor (AhR) and c-Fos expression, reducing nuclear translocation of p-p65 in bone marrow macrophages (BMMs), ultimately resulting in suppression of osteoclast resorption activity. AhR acts as a key positive regulator in the process of osteoclastogenesis, and its overexpression restored IA-mediated inhibition of osteoclast formation. *In vivo*, daily IA supplementation protected mice against OVX-induced bone loss, with higher PINP and lower CTX-1 levels. Taken together, these findings identified IA as a promising therapeutic candidate capable of suppressing osteoclastogenesis through an AhR-dependent mechanism, providing mechanistic insight and a potential strategy for the treatment of postmenopausal osteoporosis.

## Introduction

Bone remodeling sustains skeletal integrity through a continuous process in which osteoclasts resorb old bone and osteoblasts deposit new bone 1. However, pathological conditions such as aging and metabolic diseases can disrupt this equilibrium and lead to osteoporosis, a disease characterized by deterioration of the bone microarchitecture, increased fragility, and elevated fracture risk 2. Current pharmacotherapies, including bisphosphonates and selective estrogen receptor modulators, remain the cornerstone of osteoporosis management but are associated with risks of serious adverse effects, including atypical fractures and osteonecrosis of the jaw 2-4. Therefore, the development of novel therapeutic agents with improved safety profiles remains a major priority for the treatment of osteoporosis.

There is increasing evidence of strong associations between the gut microbiota, its metabolites, and metabolic diseases, including osteoporosis 5, 6. Gut microbial metabolites influence whole-body homeostasis and the functions of multiple organ systems. It has been estimated that about 35% of small molecules in human blood are produced or modified by the gut microbiota 7. Although a small number of metabolites, including short-chain fatty acids (SCFAs) 8, bile acids 9, and trimethylamine-*N*-oxide (TMAO) 10, have been shown to play important roles in host physiology, many biologically active gut microbial metabolites remain largely unexplored.

Recent research has suggested roles of tryptophan metabolites produced by the gut microbial as key factors in preserving intestinal equilibrium 11, 12. These metabolites include indole and its derivatives indole-3-propionic acid (IPA), indole-3-lactic acid (ILA), indole-3-acetic acid (IAA), indole-3-acrylic acid (IA), and indole-3-aldehyde (IAld), which can only be generated by microbial metabolism of tryptophan 13, 14. Multiple studies have shown significant decreases in levels of IA and IPA in ovariectomized (OVX) mouse models of estrogen deficiency-induced osteoporosis 15. The microbial tryptophan metabolite ILA has also been reported to be associated with the prevalence of hand osteoarthritis in two large, community-based cohorts 16. Multiomics analyses in a large cohort showed that tyrosine and tryptophan metabolism were significantly linked to identified microbiota biomarkers and to osteoporosis 17. Chen C *et al.* 18 reported that the microbial tryptophan metabolites IAA and IPA mitigated bone loss induced by OVX through the aryl hydrocarbon receptor (AhR)-mediated gut-bone pathway. Peng *et al.* 19 showed that IPA produced by *Clostridium sporogenes* in the gut inhibited osteoclast differentiation and alleviated osteoporosis-related bone loss. Taken together, these findings suggested that indole and its derivatives, derived from gut microbial tryptophan metabolism, may have beneficial roles in bone metabolic diseases.

The AhR is a highly conserved ligand-dependent transcription factor that is widely expressed in immune, epithelial, endothelial, and stromal cells of barrier tissues 20. Indole and its derivatives are classical ligands of AhR 21. Several studies have shown that AhR activation can modify innate and adaptive immune responses in a ligand-dependent manner 7, 22, 23. Under physiological conditions, AhR is bound to heat shock protein 90 (HSP90) and primarily resides in the cytoplasm. Upon ligand binding, AhR undergoes translocation into the nucleus, where it binds to the aromatic hydrocarbon receptor nuclear translocator (ARNT) to form a heterodimer that activates downstream target genes 24. Beyond this classical signaling pathway, AhR can also influence transcriptional responses through interactions with other transcription factors and coactivators, including ESR, Nrf2 and NF-κB 25.

Previous studies showed that AhR positively regulates osteoclast formation 25-27. However, the molecular mechanisms linking microbial tryptophan metabolites and osteoporosis remain unclear. In particular, the roles of AhR-mediated signaling induced by tryptophan metabolites in osteoporosis have not been fully explored.

## Methods

### Reagents and antibodies

IA (purity = 99.45%), MG-132, and dimethylsulfoxide (DMSO) were provided by MedChemExpress (Monmouth Junction, NJ, USA). Antibodies against c-Fos (#2250), GAPDH (#2118), p-p65 (#3033), p65 (#8242), JNK (#9252), p-p38 (#4511), p38 (#9212), p-AKT (#4060), and AKT (#9272) were obtained from Cell Signaling Technology (Danvers, MA, USA). Antibodies against p-ERK (#ET1610-13), ERK (#ET1601-29), p-IκBα (#ET1609-78), p-JNK (#ET1609-42) were provided by HUABIO (Hangzhou, China). Anti-MMP9 antibody (#ab283575) was provided by Abcam (Cambridge, MA, USA). Antibodies against NFATc1 (#sc-7294) and CTSK (sc-48353) were purchased from Santa Cruz Biotechnology (Santa Cruz, CA, USA). Anti-AhR antibody (#bs-21600R) was sourced from Bioss (Woburn, MA, USA).

### Isolation of bone marrow macrophages and osteoclast differentiation *in vitro*

For osteoclast differentiation *in vitro*, bone marrow macrophages (BMMs) were extracted from the bone marrow of mouse femurs and cultured in α-minimal essential medium (α-MEM) containing 10% fetal bovine serum (FBS, SA101.02; Cellmax, Beijing, China) and 30 ng/mL macrophage colony-stimulating factor (M-CSF; (MedChemExpress). To induce osteoclast differentiation, BMMs were cultured with recombinant mouse RANKL (50 ng/mL) (#AM10004; Amizona Scientific LLC) and 30 ng/mL M-CSF for indicated time.

### Mouse bone marrow mesenchymal stem cell culture and osteogenic differentiation

Mouse bone marrow mesenchymal stem cells (mBMSCs, #MUBMX-01001) were obtained from Cyagen Biosciences Inc. (Guangzhou, China) The cells were maintained in α-MEM (Abbkine, Wuhan, China) supplemented with 10% FBS. To induce osteogenic differentiation, mBMSCs were induced with osteogenic induction medium (OIM) composed of low-glucose Dulbecco's modified Eagle's medium (DMEM), 10% FBS, 100 nM dexamethasone (M2176; AbMole BioScience, Houston, TX, USA), 0.2 mM ascorbic acid, and 10 mM β-glycerophosphate (MedChemExpress).

### CCK8 assay

Cell Counting Kit-8 (CCK-8; Beijing Boxbio Science & Technology Co., Ltd., Beijing, China) assays were performed to assess the effects of IA on the viability of BMMs and BMSCs. Briefly, aliquots of 1 × 10^3^ cells were seeded onto 96-well plates with various concentrations of IA and cultured for 1, 2, or 3 days. After the indicated times, CCK-8 reagent was added to each well in accordance with the manufacturer's protocol, and the absorbance at 450 nm (A_450_) was recorded using a microplate reader (ELX808; BioTek, Winooski, VT, USA).

### TUNEL assay for detection of apoptosis

Following a 3-day treatment with IA at various concentrations, apoptosis of BMMs was evaluated by terminal deoxynucleotidyl transferase dUTP nick-end labeling (TUNEL) assay using a commercial kit (#KGA1408; Keygen BioTECH, Jiangsu, China) according to the manufacturer's instructions. TUNEL-positive cells were imaged by fluorescence microscopy (Leica Microsystems, Wetzlar, Germany).

### TRAP staining

Following osteoclast differentiation, cells were washed with phosphate-buffered saline (PBS) and fixed with 4% paraformaldehyde (PFA) for 10 min. Subsequently, they were stained using a TRAP kit (#AMK1002; Amizona Scientific LLC) in accordance with the manufacturer's protocol. Multinucleated cells with three or more nuclei were defined as TRAP-positive osteoclasts.

### Alkaline phosphatase and alizarin red S staining

After osteogenic differentiation for various times, alkaline phosphatase (ALP) staining was evaluated using a commercial kit (Beyotime Biotechnology, Shanghai, China) in accordance with the manufacturer's directions. For alizarin red S (ARS) staining, mBMSCs were fixed with 4% PFA and treated with alizarin red S (Cyagen Biosciences, Guangzhou, China) for 20 min.

### F-actin ring staining

Following osteoclast differentiation, cells were fixed with 4% PFA, permeabilized with 0.1% Triton X-100, and then blocked with 5% bovine serum albumin (BSA). The samples were then stained with phalloidin (MedChemExpress) for 30 min to visualize F-actin, and finally the nuclei were counterstained with 4′,6-diamidino-2-phenylindole (DAPI; Shandong Sparkjade Biotechnology Co., Ltd., Shandong, China) for 5 min and examined by fluorescence microscopy (Leica Microsystems).

### Bone resorption activity

BMMs were seeded on bone slices (#AMB1002; Amizona Scientific LLC). The cells were then induced to undergo osteoclast differentiation for 15 days, with the culture medium refreshed every other day. After the indicated time, the bone slices were washed with PBS and examined by scanning electron microscopy (SEM; JEOL Ltd., Tokyo, Japan). The area of resorption pits was measured in three randomly selected fields per slice using ImageJ (NIH, Bethesda, MD, USA).

### Quantitative reverse transcriptase polymerase chain reaction

Cultured cells were harvested and mRNA was isolated using RNAex Pro RNA reagent (#AG21102; ACCURATE BIOTECHNOLOGY (HUNAN) CO., LTD, ChangSha, China) according to the manufacturer's protocol, and transcribed into cDNA using a SPARKscript II RT Plus Kit (With gDNA Erase) (Shandong Sparkjade Biotechnology Co., Ltd.). Reverse transcription quantitative PCR (RT-qPCR) was performed for 40 cycles with the obtained cDNA as a template and specific primers (Table 1) using 2× SYBR Green qPCR Mix (Shandong Sparkjade Biotechnology Co., Ltd.). The data were analyzed with relative quantification based on the ΔΔCt method using GAPDH as a reference.

### Western blotting analysis

Cell lysates were prepared using RIPA lysis buffer containing phosphatase and protease inhibitors (#KGB5101-2 and KGB5101-100, Keygen BioTECH, Jiangsu, China). Equal aliquots of protein were electrophoresed on a 4%-12% gradient polyacrylamide gel (FuturePAGE™; Boyi Biotech, Changzhou, China) and transferred onto polyvinyl difluoride (PVDF) membranes. The membranes were blocked with 5% skim milk and incubated with primary antibodies overnight at 4°C. After incubation, the membranes were incubated with horseradish peroxidase (HRP)-conjugated secondary antibody for 1 h. Immunoreactive bands were detected using ECL Luminescent Solution (#KGC4601; Keygen BioTECH), visualized by chemiluminescence (Thermo Fisher Scientific, Waltham, MA, USA), and quantified using ImageJ (NIH).

### Immunofluorescence

Following 5 days of stimulation with or without RANKL, BMMs were fixed, permeabilized, and incubated overnight with anti-NFATc1 antibody (#K002822P; Solarbio Life Sciences, Beijing, China), followed by a 60-min incubation with FITC- or TRITC-conjugated secondary antibody. After nuclear counterstaining with DAPI, the samples were visualized by confocal microscopy.

### Lentiviral transfection

Recombinant lentiviral vector particles encoding mouse AhR were custom ordered from HyCyte (Suzhou, China). For transduction experiments, the mouse BMM complete medium was exchanged for 2% FBS containing M-CSF (30 ng/mL), followed by addition of lentiviral particles at 150 MOI and 5 μg/mL polybrene (HyCyte). The infection medium was exchanged for fresh growth medium after 18 h of transfection. Nontransfected cells were eliminated by selection with puromycin (4 μg/mL; MedChemExpress).

### RNA interference

Gene silencing was performed by transfecting cells with specific AhR-targeting small interfering RNAS (siRNAs) or negative control (NC) using NanoTrans™ Transfection Reagent Plus in Opti-MEM (CYTOCH, Shanghai, China) in accordance with the manufacturer's instructions. The targeting sequences were: siRNA-1 GGUCCGAAGCACACGCAAATT; siRNA-2 UUUGCGUGUGCUUCGGACCTT.

### Coimmunoprecipitation assay

Following a 4-h treatment with 0.5 mM MG132, BMMs were lysed and precleared with Protein A/G magnetic beads (MedChemExpress). They were subsequently immunoprecipitated by incubation with target-specific antibodies overnight at 4 °C, followed by addition of Protein A/G beads for 2 h. The beads were washed extensively with PBS, and the coprecipitated proteins were analyzed by Western blotting.

### 16S rRNA gene sequencing

Fecal samples were collected and genomic DNA was extracted using a commercial kit according to the manufacturer's instructions. The integrity and concentration of the extracted DNA were verified by agarose gel electrophoresis and spectrophotometry (Majorbio Bio-Pharm Technology Co., Ltd.). The hypervariable V3-V4 regions of the bacterial 16S rRNA gene were amplified with 338F_806R primers (Table 1). PCR products were purified and quantified. Sequencing libraries were constructed following the standard protocol provided by Majorbio Bio-Pharm Technology Co., Ltd. (Shanghai, China). High-throughput sequencing was performed on the Illumina MiSeq platform to generate paired-end reads (Illumina, San Diego, CA, USA). The raw sequencing data were processed using the Majorbio cloud platform.

### Targeted quantification of tryptophan metagenomic sequencing

Targeted metabolomic profiling of tryptophan metabolites in serum from Sham and OVX mice was conducted at Majorbio Bio-Pharm Technology Co., Ltd. using an Agilent 8890B/5977B GC-MS system (Agilent Technologies, Santa Clara, CA, USA). Data acquisition and peak integration were performed with Agilent MassHunter software (v10.0.707.0), generating a quantitative data matrix for subsequent statistical analysis. The correlations between two variables were examined by Spearman's correlation analysis.

### RNA sequencing

BMMs were induced to undergo osteoclast differentiation by incubation with IA or vehicle alone for 4 days. All RNA sequencing (RNA-seq) processes were performed by Majorbio Bio-pharm Biotechnology Co., Ltd.

### Molecular docking

To investigate the potential binding of IA to the AhR, a blind docking study was conducted using the CB-Dock2 server (https://cadd.labshare.cn/cb-dock2/index.php). The structures of the ligand (IA, PubChem CID: 5375048) and receptor (AhR, PDB ID: 5NJ8, 3.3 Å resolution) were acquired from the respective databases 28. The docking procedure integrated automated cavity prediction, docking simulation, and template fitting.

### Animal experiments

All animal experiments were conducted in accordance with the ARRIVE guidelines and were approved by the Ethics Committee of Peking University Third Hospital. Eight-week-old female C57BL/6 mice were obtained from the Medical Laboratory Animal Center of Peking University. After 1 week of acclimatization, 24 mice were randomly assigned to 4 groups: Sham group, OVX control group, OVX + IA (20 mg/kg) group, and OVX + IA (40 mg/kg) group. The OVX model was established via a dorsal approach by surgically removing the bilateral ovaries. In the Sham group, the ovaries were exposed but not excised before suturing. Starting 1-week after surgery, mice were administered IA or an equal volume of normal saline by daily gavage for 7 weeks. Serum and femur samples were collected at the end point (8 weeks post-surgery) for subsequent analysis.

### Micro-CT examination and analysis

Isolated mouse femurs were fixed in 4% PFA and imaged using a Skyscan 1276 micro-CT scanner (Bruker, Billerica, MA, USA) at 6-μm resolution (50 kV, 200 μA). Image reconstruction and analysis were conducted using Bruker's software suite (Nrecon, DataViewer, CTan, CTvox). Trabecular bone structure was evaluated within a region of interest (ROI) 1.0 mm high starting from the growth plate. The 3D reconstructions revealed abundant trabeculae, and standard microarchitecture parameters such as BV/TV, Tb.N, Tb.Th and Tb.Sp were measured and analyzed.

### Histological analysis

Mouse femurs were decalcified after 2 days of fixation with 4% PFA. The femurs were then embedded in paraffin and cut into sections 5-7 μm thick for histological analysis. Tissue sections were subjected to hematoxylin and eosin (H&E) and TRAP staining. Osteoclasts were quantified as TRAP-positive multinucleated cells exceeding 200 µm in diameter on microscopic images using ImageJ (NIH).

### ELISA

Serum biomarkers were measured using specific ELISA kits following the manufacturers' instructions. Specifically, P1NP and CTX-1 levels were assessed with kits (#E-EL-M3023 and #E-EL-M0233, respectively) from Elabscience Biotechnology (Wuhan, China), while ALT and AST levels were determined using an ALT kit and AST kit (#SU-BN27830 and #SU-BN21534, respectively; Chengzhikewei Biotechnology, Beijing, China).

### Statistical analysis

All data are expressed as the mean ± standard deviation. All statistical analyses were performed using GraphPad Prism (v8.0; GraphPad Software, San Diego, CA, USA). Differences between two groups were assessed by the unpaired Student's *t* test. For comparisons among three or more groups, one-way ANOVA was applied, followed by Tukey's post hoc test. In all analyses, *P* < 0.05 was taken to indicate statistical significance.

## Results

### Estrogen deficiency-induced osteoporosis led to alterations in gut microbiota and reduction of tryptophan metabolites

**Figure 1:**
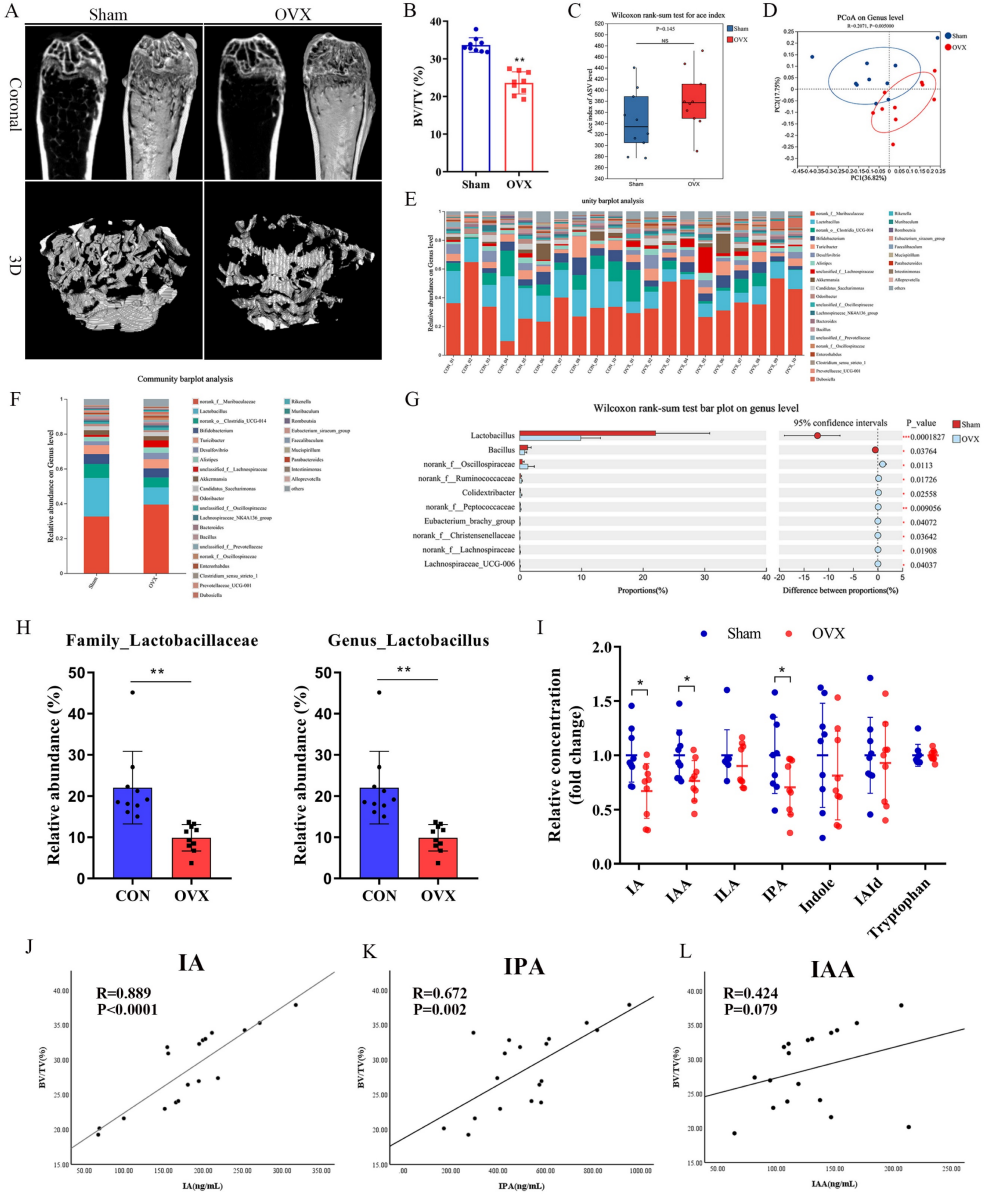
Estrogen deficiency-induced osteoporosis led to alterations in gut microbiota and reduction of tryptophan metabolites. (A, B) Representative micro-CT images of distal femur and quantification of trabecular bone volume per tissue volume (BV/TV), n=9 per group, Scale bar: 100μm. (C) α-diversity analysis based on Wilcoxon rank-sum test. (D) β-diversity analysis based on PCoA. (E, F) Relative abundances of the gut microbiota at the genus levels. Each column represents a sample; Each column represents a group. (G) Wilcoxon rank-sum test about gut microbiota at the genus level between two groups. (H) Relative abundance of family_Lactobacillaceae and genus_Lactobacillaceae. (I) The concentration of indole and its derivatives in serum based on targeted tryptophan metabolomics sequencing. n = 9. (J, K, L) Spearman correlation analysis between the BV/TV and microbial tryptophan metabolites (IA, IPA and IAA).

The OVX mouse model was confirmed to show significant bone loss after 8 weeks 2. In the present study, bone volume per tissue volume (BV/TV) was markedly reduced in the OVX group compared to the Sham group (Fig. 1A, B). To examine whether OVX-induced bone loss was associated with alterations in the gut microbiota, we performed 16S rRNA gene sequencing in fecal samples. While the ACE index showed a nonsignificant trend in α-diversity (Fig. 1C), β-diversity assessed by principal coordinates analysis (PCoA) based on UniFrac distance revealed significant separation between the OVX and Sham groups (*P* = 0.005) (Fig. 1D). In addition, the gut microbiome health index (GMHI) was significantly decreased, while the microbial dysbiosis index (MDI) index was significantly increased in the OVX group compared with the Sham group (Fig. S1A, B). Taxonomic analysis further demonstrated marked alterations in microbiota composition (Fig. S1C). At the genus level, the OVX group showed significant reductions in the abundance levels of *Lactobacillus* and *Clostridium*, accompanied by significant increases in *Muribaculaceae* and *Lachnospiraceae* (Fig. 1E, F). The family Lactobacillaceae and genus *Lactobacillus* showed particularly notable downregulation (Fig. 1H), with significant differences at the genus level between groups identified by Wilcoxon's rank-sum test (Fig. 1G). Taken together, these observations indicated that OVX-induced osteoporosis remodeled the gut microbial composition, significantly altering its diversity and community composition.

*Lactobacillus* is the dominant bacterial genus in the gut responsible for producing ILA, IAA, and IA 29-31, while *C. sporogenes* is the main producer of IPA 32, 33. Tryptophan metabolism has been shown to play an important regulatory role in bone homeostasis. Indoles and their derivatives represent a unique class of tryptophan metabolites that are produced exclusively by gut microbiota 7. These indoles and their derivatives have attracted increasing attention due to their beneficial roles in both the intestine and in extraintestinal organs 11. Targeted metabolomics analyses confirmed that serum levels of IA, IAA, and IPA were significantly lower in the OVX group compared to the Sham controls (Fig. 1I). Consistent with previous reports, IA and IAA were significantly positively correlated with the relative abundance of *Lactobacillus* (Fig. S1H, I). Spearman's correlation analysis revealed significant positive correlations of both IA and IPA with BV/TV (Fig. 1J, K), while other indole derivatives (IAA, IAld, ILA, indole, and tryptophan) showed no such correlations (Fig. 1L and S1D-G). IPA and IAA have been reported to be related to OVX-induced bone loss. Peng *et al.* 19 showed that gut *C. sporogenes*-derived IPA suppresses osteoclast formation by activating pregnane X receptor. Our results showed that IA level was significantly decreased in OVX mice, which showed the strongest positive correlation with BV/TV. As its function and mechanisms of action in osteoporosis remain largely unexplored, IA represents a novel and promising candidate for further investigation.

### Indoleacrylic acid inhibited RANKL-induced osteoclast formation and bone resorption

**Figure 2:**
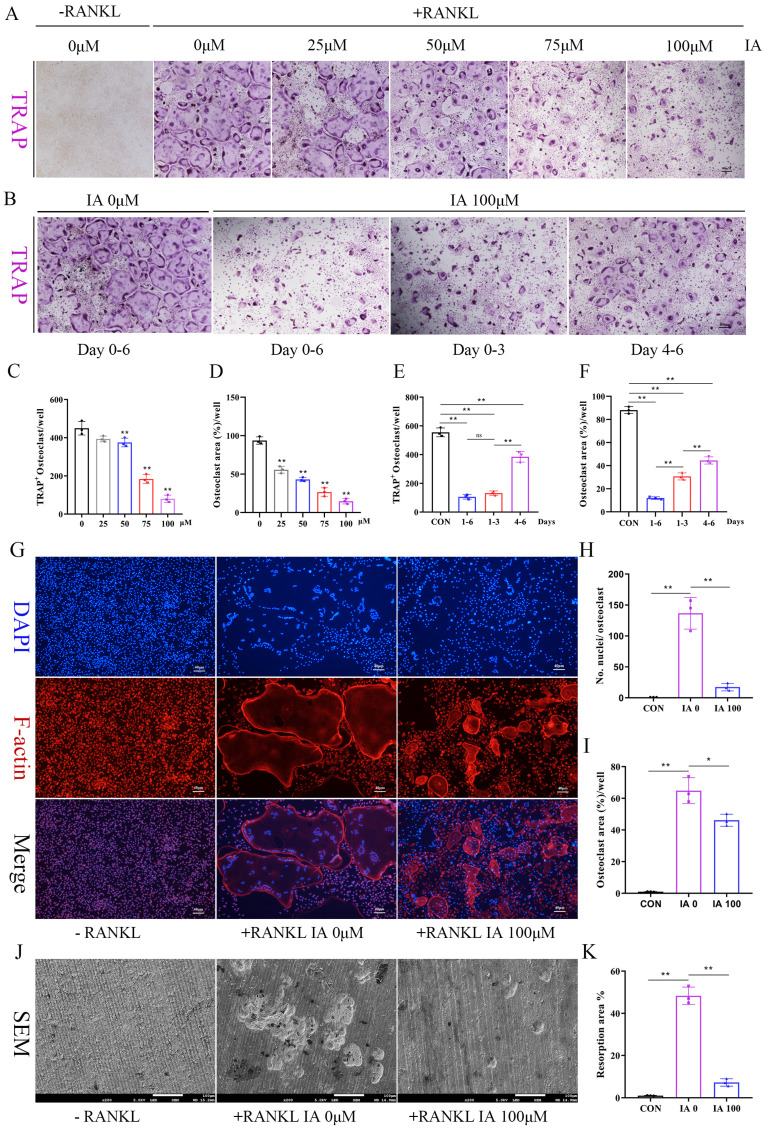
IA inhibited RANKL-induced osteoclast formation and bone resorption. (A) Representative image of TRAP staining showing that IA inhibited osteoclastogenesis dose-dependently with BMMs. scale bar, 100 µm; (B) Representative images of TRAP staining showing BMMs treated with IA (100μM) for the indicated days during osteoclastogenesis. scale bar, 100 µm; (C, E) Quantification of the TRAP-positive multinucleated cells (nuclei > 3). (D, F) Quantification of the TRAP-positive area of osteoclast (%). (G) The F-actin (Red) and nuclei (blue) staining of osteoclasts were photographed by fluorescence microscope. scale bar, 20 µm; (H, I) Quantification of the nuclear aggregation number per osteoclast and relative number of osteoclasts. (J) Representative images of SEM scanning showing bone resorption function of BMMs with or without IA (100μM). scale bar, 50 µm; (K) Quantification of the resorption area (%). Data are shown as mean±SD, n = 3, *p < 0.05 **p < 0.01.

Increased osteoclast number and activity are key pathogenic features of postmenopausal osteoporosis 34. To explore IA's role in osteoclast differentiation, BMMs were cultured with various concentrations of IA (0-100 μM) in the presence of RANKL. Concentration-dependent inhibition of osteoclastogenesis by IA was observed by TRAP staining, with nearly complete suppression at 100 μM IA (Fig. 2A, C, D). To identify the stage at which it exerted its inhibitory effects, BMMs were treated with 100 μM IA at different time points. The results showed that IA inhibited osteoclast differentiation at both early (1-3 days) and mid-late stages (3-6 days), with the strongest inhibitory effect in the early stage (Fig. 2B, E, F). CCK-8 and TUNEL assays showed that IA had no impact on BMM proliferation or apoptosis, excluding these processes as potential mechanisms for its inhibitory effect on osteoclast formation (Fig. S2A-C).

Furthermore, morphological analyses showed that IA markedly reduced osteoclast size and multinucleation, as determined by F-actin/DAPI co-staining. This was accompanied by potent inhibition of F-actin ring formation (Fig. 2G-I). Consistent with these findings, bone resorption assays revealed a reduction in resorptive activity, with the resorbed area decreasing from 48.3% in the control group to 7.2% in the 100 μM IA treatment group (Fig. 2J, K).

### Indoleacrylic acid inhibited osteoclast-related markers and NFATc1 nuclear translocation

**Figure 3:**
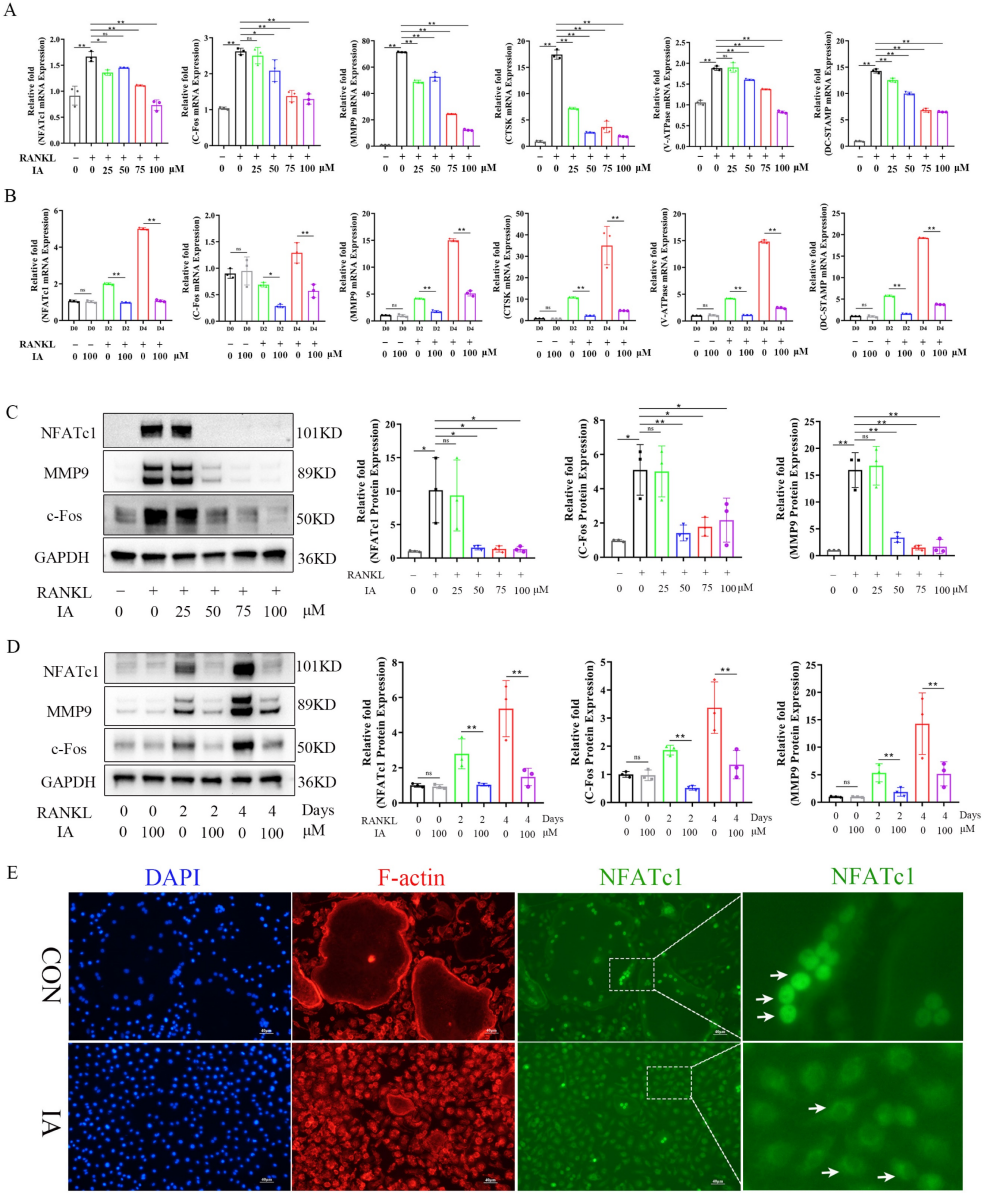
IA inhibited osteoclast-related markers and NFATc1 nuclear translocation. (A) mRNA expression levels of Nfatc1, c-fos, Ctsk, V-atpase and Dc-Stamp with various concentration of IA intervention after 2 days. (B) mRNA expression levels of Nfatc1, c-fos, Ctsk, V-atpase and Dc-Stamp after various times with IA (100μM) intervention. (C) Western blot for the expression of NFATC1, MMP9 and c-Fos in RANKL-induced BMMs with various concentration of IA. (D) Western blot for the expression of NFATC1, MMP9 and c-Fos with various time with IA (100μM) intervention. (E) Representative fluorescence images of NFATc1 in the presence of IA (0, 100μM). Scale bar = 50μm. Data are presented as means ± SD of 3 independent experiments; *p < 0.05 **p < 0.01, ns: no significant differences.

The master transcription factors c-Fos and NFATc1 orchestrate osteoclastogenesis by upregulating key marker genes, including *CTSK*, *MMP9*, and *V-ATPase* 35. RT-qPCR was performed to measure the expression of these genes. The results demonstrated that IA dose-dependently suppressed the expression of *Nfatc1* and *c-fos*, as well as the osteoclast-related genes *Mmp9*, *Ctsk*, *V-atpase*, and *Dc-stamp* (Fig. 3A). This suppression was consistent across various stages of osteoclast differentiation (Fig. 3B). Consistent with these findings, western blotting analysis confirmed that the levels of NFATc1, c-Fos, and MMP9 protein were also significantly reduced by IA during osteoclast differentiation (Fig. 3C, D), and immunofluorescence staining showed that IA prevented the RANKL-induced nuclear translocation of NFATc1 (Fig. 3E). Taken together, these observations established that IA suppressed osteoclastogenesis by repressing RANKL-induced osteoclast-specific gene and protein expression.

Next, we used mBMSCs to examine the effects of IA on osteoblastic differentiation. IA showed no effects on proliferation of mBMSCs (Fig. S3A) or the mRNA expression of key osteogenic markers, including *Runx2*, *Sp7*, *Ocn*, and *Opn* (Fig. S3B). Functional assays confirmed that IA had no significant effect on early osteogenic differentiation, as shown by ALP staining and activity (Fig. S3C, D), nor on late-stage mineralization, as quantified by ARS staining (Fig. S3E, F). Taken together, these results indicated that IA did not directly influence osteoblastic differentiation.

### Indoleacrylic acid suppressed RANKL-induced activation of the NF-κB pathway but not the MAPK pathway

**Figure 4:**
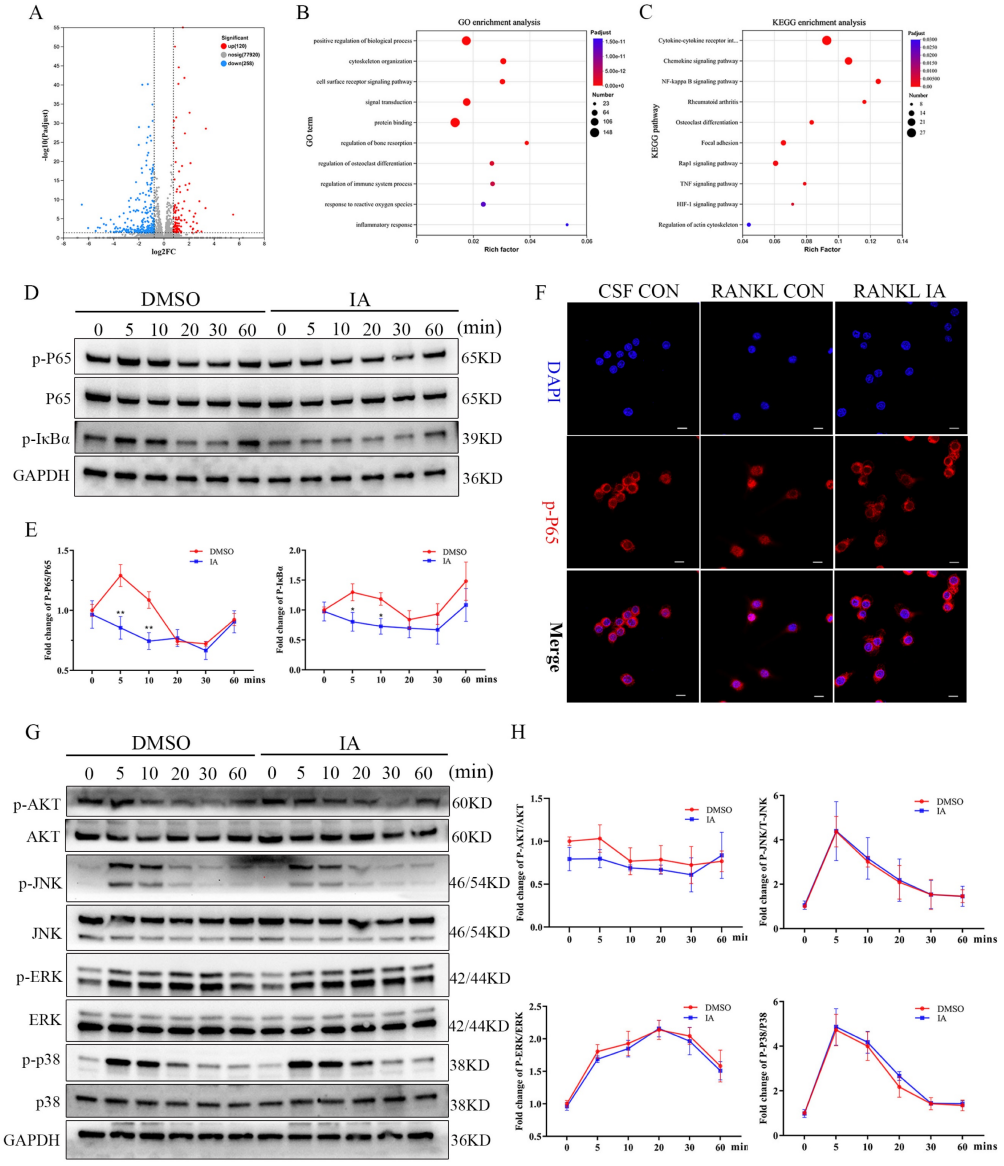
IA suppressed RANKL-induced activation of the NF-κB pathway but not the MAPK pathway. (A) Volcano plot showing fold change and statistical significance of each gene calculated by differentially expressed genes (DEGs) analysis. (B) GO terms in biological process were used for functional enrichment clustering analysis on common DEGs. (C) KEGG enrichment analysis showed various signaling pathways were significantly altered after IA treatment. (D) BMMs were pretreated with IA (0, 100μM) for various time periods (0, 5, 10, 20, 30 and 60 min) after RANKL induced osteoclast differentiation. Western blot for the expression of p-P65, P65 and p-IκBα. (E)The ratios of p-IκB-α to GAPDH bands, p-P65 bands to total P65 bands were determined using Image J. (F) Representative fluorescence images of p-P65 in the presence of IA (0, 100μM) for 10min after RANKL induced osteoclast differentiation. Scale bar = 10μm. (G) Western blot for the expression of p-AKT, AKT, p-JNK, JNK, p-ERK, ERK, p-P38 and P38. (H) The ratios of p-AKT, p-JNK, p-ERK, p-P38 bands to total AKT, JNK, ERK, P38 bands were determined using Image J. Data are presented as means ± SD of 3 independent experiments; *p < 0.05 **p < 0.01, ns: no significant differences.

To elucidate further the mechanism of IA-mediated inhibition of osteoclastogenesis, BMMs stimulated with RANKL in the presence or absence of IA were subjected to RNA-seq and bioinformatics analyses. The heat map identified 378 differentially expressed genes (DEGs) with a change in expression of at least 1.8-fold in the IA treatment group (Fig. S4A). The volcano plot showed that 120 genes were upregulated and 258 were downregulated (Fig. 4A). Among these genes, 15 osteoclast-specific genes showed markedly reduced expression in the IA treatment group compared with the controls, confirming that IA successfully inhibited RANKL-induced osteoclast differentiation (Fig. S4B).

Gene Ontology (GO) enrichment analysis showed that IA influences pathways associated with cytoskeleton organization, regulation bone resorption, osteoclast differentiation, and response to reactive oxygen species. These processes are essential for the maturation of BMMs into large, multinucleated osteoclasts (Fig. 4B). Kyoto Encyclopedia of Genes and Genomes (KEGG) analysis revealed significant enrichment of NF-κB, Rap1 signaling, TNF signaling, and actin cytoskeleton regulation pathways, all of which play critical roles in osteoclast differentiation (Fig. 4C). Gene set enrichment analysis (GSEA) confirmed significant decreases in the expression of genes associated with the NF-κB signaling pathway following treatment with IA (Fig. S4C).

To validate these findings, we examined the effects of IA on key RANKL-activated signaling pathways. Western blotting analysis revealed that IA (100 μM) attenuated the phosphorylation of IκBα and p65 in a time-dependent manner (0-60 min). Immunofluorescence analysis confirmed that IA significantly suppressed the nuclear translocation of p65 (Fig. 4F). Conversely, IA did not markedly affect early phosphorylation events in the MAPK pathway, including ERK, JNK, and p38, and had no effect on PI3K-AKT signaling in response to RANKL stimulation (Fig. 4G, H). Taken together, these results demonstrate that IA suppresses osteoclast differentiation primarily by inhibiting RANKL-induced activation of the NF-κB signaling pathway, without significantly affecting MAPK or PI3K-AKT pathways.

### Indoleacrylic acid inhibited AhR expression in bone marrow macrophages after RANKL-induced osteoclast differentiation

**Figure 5:**
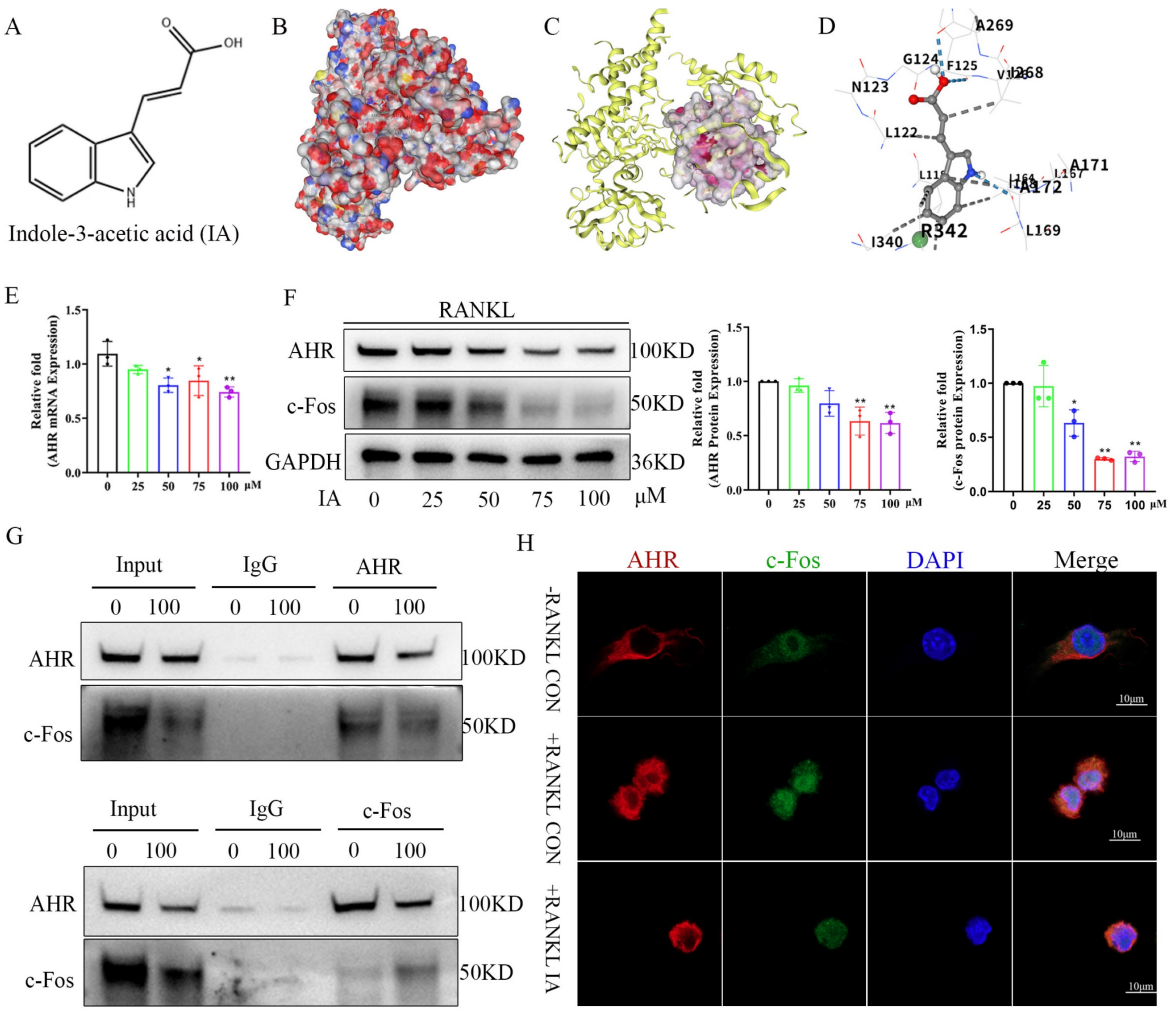
IA inhibited AhR expression in bone marrow macrophages after RANKL-induced osteoclast differentiation. (A) Schematic chemical structure of IA. (B) The space-filling model suggested that IA was buried within the ligand binding domain. (C) Molecular docking of IA with AHR was performed. (D) The hydrogen bonds and hydrophobic bond of IA to AHR were presented as Molecular docking. (E) mRNA expression levels of Ahr with various concentration of IA intervention after 4 days. (F) protein expression levels and quantification of AHR and c-Fos with various concentration of IA intervention after 4 days. (G) Coimmunoprecipitation validation of the interaction between TXNDC9 and HSP90 in BMMs. (H) Immunofluorescence colocalization of AHR and c-Fos in BMMs after RANKL induced osteoclast differentiation with or without IA. scale bar, 10 µm; Data are presented as means ± SD of 3 independent experiments; *p < 0.05 **p < 0.01, ns: no significant differences.

IA (Fig. 5A) is a well-characterized AhR ligand. Molecular docking studies were carried out to explore the binding configuration between IA and AhR. IA exhibited high binding affinity with AhR (-6.5 kcal/mol), as shown in the space-filling docking model (Fig. 5B), with hydrogen bonding interactions between ligand and receptor (Fig. 5C). Specifically, the amino group of IA formed a hydrogen bond with IIe168 of AhR, and the carboxylate group formed hydrogen bonds with Phe125 and Ala269. Eight additional hydrophobic interactions also contributed to stabilization of the IA -AhR interaction (Fig. 5D). These findings suggested that AhR is a direct molecular target of IA.

Next, we measured the expression of AhR in BMMs following IA treatment. Marked concentration-dependent decreases were observed in AhR gene and protein expression following IA treatment (Fig. 5E, F). AhR is a direct transcriptional activator of *c-Fos* 36. To explore the possible interaction between AhR and c-Fos, coimmunoprecipitation (co-IP) experiments were performed using antibodies against AhR and c-Fos. The anti-AhR antibody pulled down c-Fos protein in BMMs and, reciprocally, the anti-c-Fos antibody captured AhR indicating the interaction of these two proteins in BMMs (Fig. 5G). Dual immunofluorescence staining of AhR (red) and c-Fos (green) provided clear evidence of their colocalization in a diffuse perinuclear pattern in BMMs. IA treatment reduced the fluorescence intensity of both proteins, consistent with IA-mediated inhibition of AhR and c-Fos expression (Fig. 5H). These observations were consistent with the expression data shown in Fig. 5F.

### AhR is a critical positive regulator of osteoclastogenesis

As IA downregulated AhR expression, and previous studies showed that AhR^-/-^ mice exhibit higher bone mass and reduced bone resorption 26, 27, 37, we further explored the effect of AhR in regulation of osteoclastogenesis. First, we assessed the expression dynamics of AhR during RANKL-induced osteoclast differentiation. Both AhR mRNA and protein levels were upregulated in BMMs following stimulation with RANKL/M-CSF, which was concomitant with the induction of the key osteoclastogenic transcription factors NFATc1 and c-Fos (Fig. S5A-C). Immunofluorescence analysis showed that AhR expression increased significantly after 4 days of osteoclast differentiation (Fig. S5D, E). Moreover, analysis of the femurs of both Sham and OVX mice also indicated higher AhR expression in the OVX group, further suggesting a role for AhR in regulation of osteoclastogenesis.

**Figure 6:**
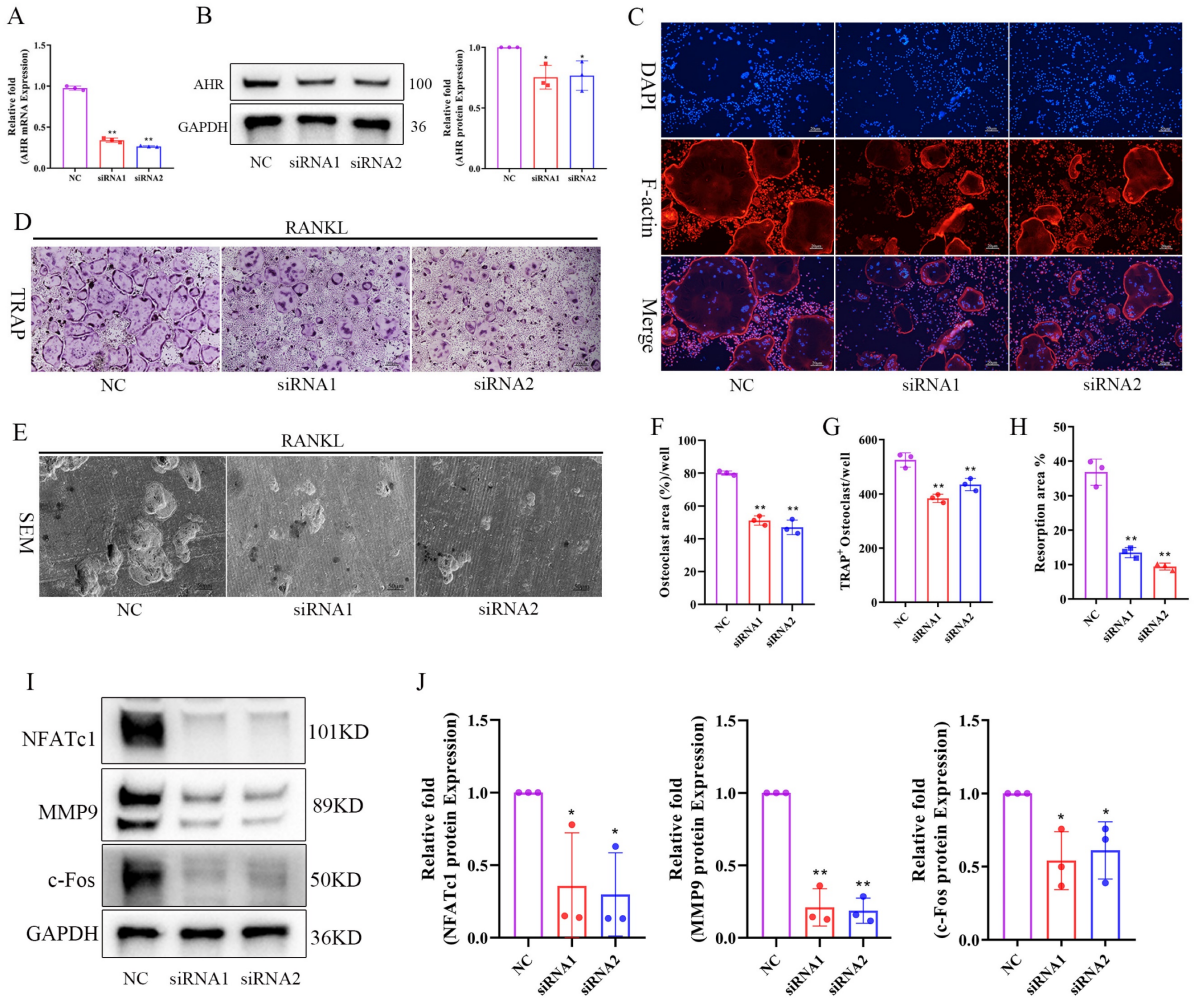
Knockdown of AHR inhibited RANKL induced osteoclast differentiation and bone resorption. (A) The knockdown efficiency of AHR mRNA by siRNA in BMMs. (B) The expression and quantification of AHR protein by siRNA in BMMs. (C) The F-actin (Red) and nuclei (blue) staining of osteoclasts were photographed by fluorescence microscope after 5 days osteoclast differentiation. scale bar, 20 µm; (D) Representative image of TRAP staining showing that knockdown of AHR inhibited osteoclastogenesis after 5 days osteoclast differentiation. scale bar, 100 µm; (E) Representative images of SEM scanning showing bone resorption function of BMMs after siRNA. scale bar, 50 µm; (F) Quantification of the TRAP-positive area of osteoclast (%). (G) Quantification of the relative number of osteoclasts. (H) Quantification of the resorption area (%). (I, J) The expression and quantification of osteoclast related proteins by western blot after 4 days osteoclast differentiation. Data are shown as mean±SD, n = 3, *p < 0.05 **p < 0.01.

Next, we performed siRNA-mediated knockdown experiments to verify the functional role of AhR. The knockdown efficiency was confirmed by RT-qPCR and western blotting analysis, with siRNA-1 and siRNA-2 markedly suppressing AhR expression at both the mRNA and protein levels (Fig. 6A, B). AhR knockdown significantly inhibited osteoclast formation (Fig. 6C, D), resulting in fewer and smaller TRAP-positive cells (Fig. 6F, G). Bone resorption activity was similarly reduced in AhR-deficient cells (Fig 6E, H). Western blotting analysis further revealed that AhR knockdown decreased the expression of NFATc1, c-Fos, and MMP9 in BMMs (Fig. 6I, J). These findings demonstrated that AhR functions as a critical positive regulator of osteoclastogenesis, with its suppression inhibiting both osteoclast formation and resorption activity.

### AhR overexpression ameliorated the inhibition of indoleacrylic acid on osteoclast formation

**Figure 7:**
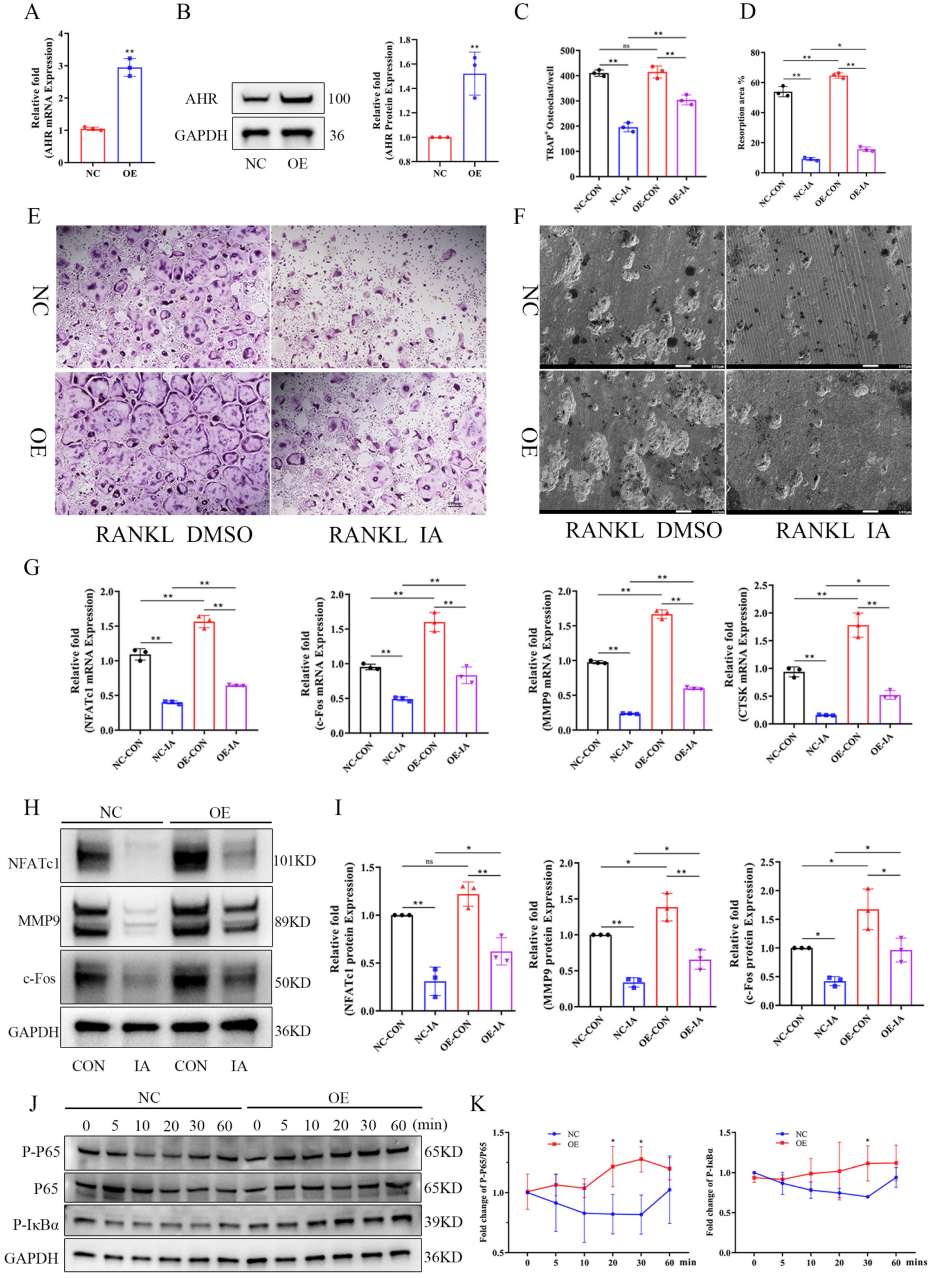
AhR overexpression ameliorated the inhibition of IA on osteoclast formation. (A) The over-expression efficiency of AHR mRNA by lentivirus transfection in BMMs. (B) The expression and quantification of AHR protein by lentivirus transfection in BMMs. (C) Quantification of the relative number of osteoclasts. (D) Quantification of the resorption area (%). (E) Representative image of TRAP staining showing that overexpression of AHR promoted osteoclastogenesis after 5 days osteoclast differentiation. scale bar, 100 µm; (F) Representative images of SEM scanning showing bone resorption function of BMMs after lentivirus transfection. scale bar, 50 µm; (G) mRNA expression levels of Nfatc1, c-fos, Ctsk and Mmp9 in BMMs by lentivirus transfection. (H) The expression and quantification of osteoclast related proteins by western blot. (G) Western blot for the expression of p-P65, P65 and p-IκBα in BMMs after lentivirus transfection. Data are shown as mean±SD, n = 3, *p < 0.05 **p < 0.01.

Given that AhR positively regulated osteoclastogenesis and that IA treatment downregulated AhR expression, we postulated that AhR mediates the inhibitory effect of IA on osteoclast differentiation. To test this hypothesis, we generated AhR-overexpressing (OE) BMMs using lentiviral transduction. The overexpression efficiency of AhR by lentivirus transfection was confirmed by RT-qPCR and western blotting analysis, both of which showed markedly elevated AhR expression (Fig. 7A, B). NC and AhR-OE BMMs were then induced to undergo RANKL-mediated osteoclast differentiation with or without IA (100 μM). The results showed that AhR overexpression indeed increased osteoclast formation and bone resorption compared with NC cells (Fig. 7C-F). However, the inhibitory effect of IA was rescued by AhR overexpression, which increased both osteoclast function (Fig. 7C, E) and bone resorption activity (Fig. 7D, F). Consistent with these functional results, IA reduced the expression of osteoclast-specific genes and proteins, including NFATc1, MMP9, c-Fos, and CTSK, in NC BMMs. In contrast, the IA-induced suppression of these markers was significantly alleviated in AhR-OE BMMs (Fig. 7G-I).

Because IA inhibited NF-κB signaling after RANKL-induced osteoclast differentiation in BMMs, we next examined whether AhR overexpression influenced activation of NF-κB signaling. Western blotting analysis demonstrated increased phosphorylation of p65 and IκBα in AhR-OE BMMs (Fig. 7J, K), suggesting that AhR promoted osteoclast differentiation by activating NF-κB signaling and that AhR overexpression counteracted the inhibitory effects of IA.

### Indoleacrylic acid supplementation protected mice against OVX-induced bone loss

**Figure 8:**
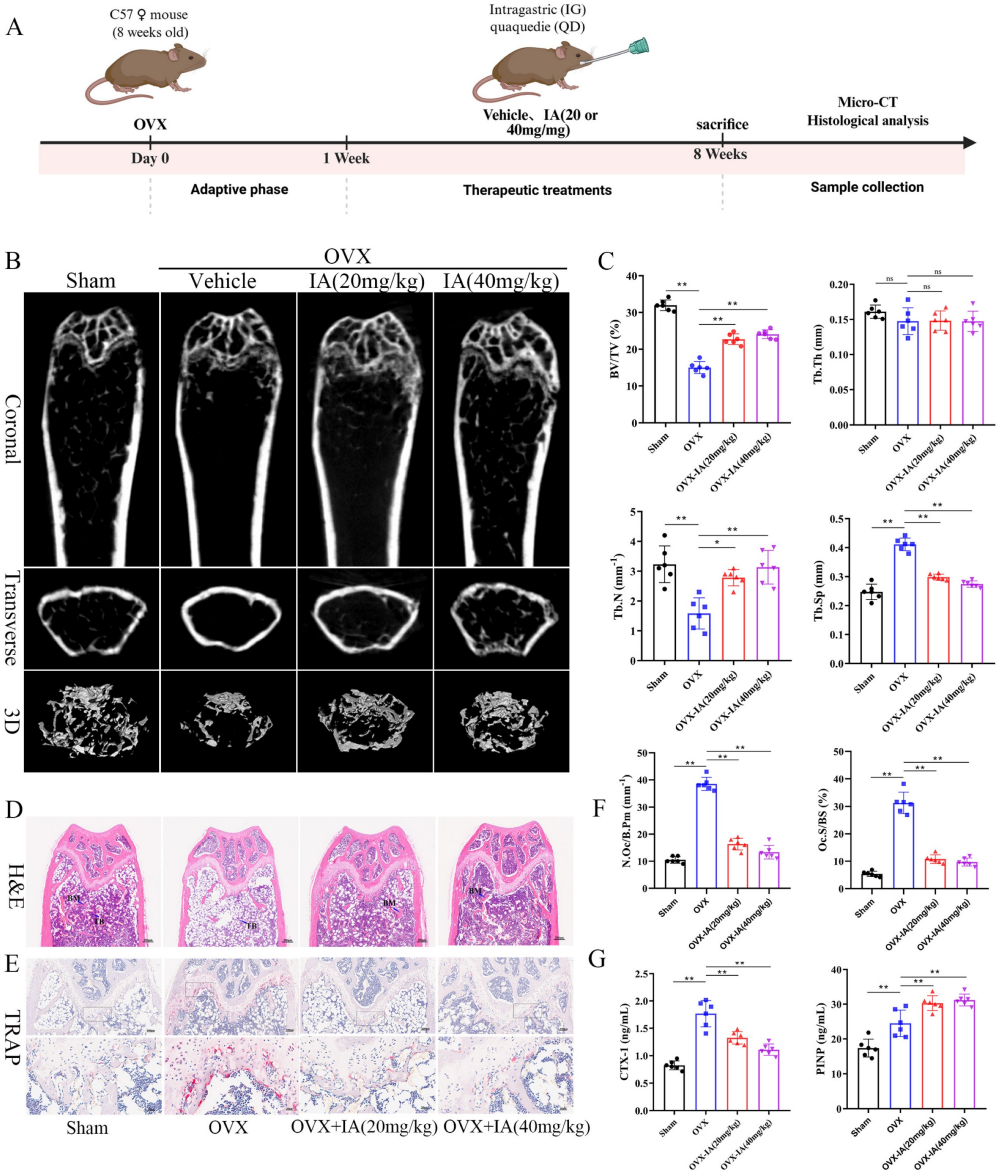
IA supplementation protected mice against OVX-induced bone loss. (A) Schematic illustration of the experimental steps involved in this study. (B) Representative micro-CT images of trabecular (Tb) and cortical (Ct) bone from four groups (n = 6 per group, Scale bar; Tb 100 μm, Ct 1 mm). (C) Quantification of BV/TV, Tb.N, Tb.Th and Tb.Sp in femur tissues. (D) Representative pictures in H&E staining of distal femoral sections. (E) Representative pictures in TRAP stained (scale bar, 100 µm;). (F) N.Oc/B.Pm and Oc.S/BS of osteoclasts from slices of distal femur was evaluated in each group in two different fields of view. (G) The levels of serum CTX-I and PINP among the groups. Data are presented as means ± SD. (n = 6) *p < 0.05 **p < 0.01, ns: no significant differences.

Given the reduced serum IA levels observed in OVX mice and its inhibitory effects on osteoclastogenesis *in vitro*, we evaluated the therapeutic potential of IA in an OVX-induced osteoporosis model. Ovariectomized mice were administered IA (20 or 40 mg/kg) by daily gavage for seven weeks. The experimental timeline is illustrated schematically in Fig. 8A. IA supplementation significantly attenuated OVX-induced bone loss, as demonstrated by increased BV/TV and Tb.N and decreased Tb.Sp relative to OVX controls (Fig. 8B, C). As expected, OVX mice exhibited significant body weight gain and uterine atrophy compared with Sham mice, confirming successful model establishment (Fig. S6A, B). IA treatment showed a modest trend toward reduced body weight, although this change was not statistically significant (Fig. S6A). Importantly, IA did not produce detectable toxicity, as indicated by unchanged serum ALT and AST levels (Fig. S6C) and the absence of histopathological abnormalities in major organs (Fig. S6D). Histological analyses further supported the protective effects of IA. TRAP staining showed a marked increase in osteoclast numbers along the trabeculae in OVX mice, which was substantially reduced following IA treatment (Fig. 8E, F). Consistently, H&E staining revealed severe trabecular bone loss in OVX mice, whereas IA supplementation restored trabecular density and thickness in the distal femoral metaphysis (Fig. 8D).

Serum bone turnover markers were next measured to assess systemic bone metabolism. OVX significantly elevated expression of the bone resorption marker CTX-I, accompanied by a compensatory increase in the bone formation marker PINP, reflecting high bone turnover (Fig. 8G). IA treatment normalized this imbalance, producing a dose-dependent reduction in CTX-I. In addition, IA further increased PINP levels above the OVX baseline, suggesting a shift toward improved bone formation (Fig. 8G). Taken together, these *in vivo* findings showed that IA mitigated OVX-induced bone loss largely through the suppression of osteoclast activity and bone resorption, with evidence of concurrent enhancement of bone formation.

## Discussion

Osteoporosis is an age-related bone metabolic disease, and the gut microbiota and their metabolites are increasingly recognized as important regulators of bone homeostasis 7. The results of the present study revealed significant changes in the gut microbial composition in an OVX mouse model of osteoporosis, including a significant decrease in the GMHI. Notably, the abundance levels of *Lactobacillus* and *Clostridium* were significantly decreased in OVX mice. Previous studies have shown that bacteria belonging to these genera are key contributors to tryptophan-derived metabolites such as indole and its derivatives 18, 19. Consistent with these findings, the levels of IA, IAA, and IPA were significantly decreased in the OVX group in the present study. Spearman correlation analysis confirmed that IA and IAA were positively associated with the abundance of *Lactobacillus*, and IA showed the strongest correlation with bone mass. Mechanistically, we found that IA significantly inhibited RANKL-induced osteoclast differentiation and bone resorption activity in BMMs. IA significantly suppressed activation of the NF-κB signaling pathway, as indicated by decreased phosphorylation and nuclear translocation of p65. As AhR is a known ligand-binding receptor for IA, we examined its involvement in this process. Treatment with IA significantly downregulated AhR expression in BMMs. AhR interacted with c-Fos, a key transcription factor required for osteoclastogenesis. Previous studies 25, 27 and our own findings showed that AhR is an important positive regulator of osteoclast differentiation. We found that AhR expression was significantly increased during RANKL-induced osteoclastogenesis and in the bones of OVX mice, whereas siRNA-mediated knockdown of AhR significantly inhibited RANKL-induced osteoclastogenesis.

**Figure 9:**
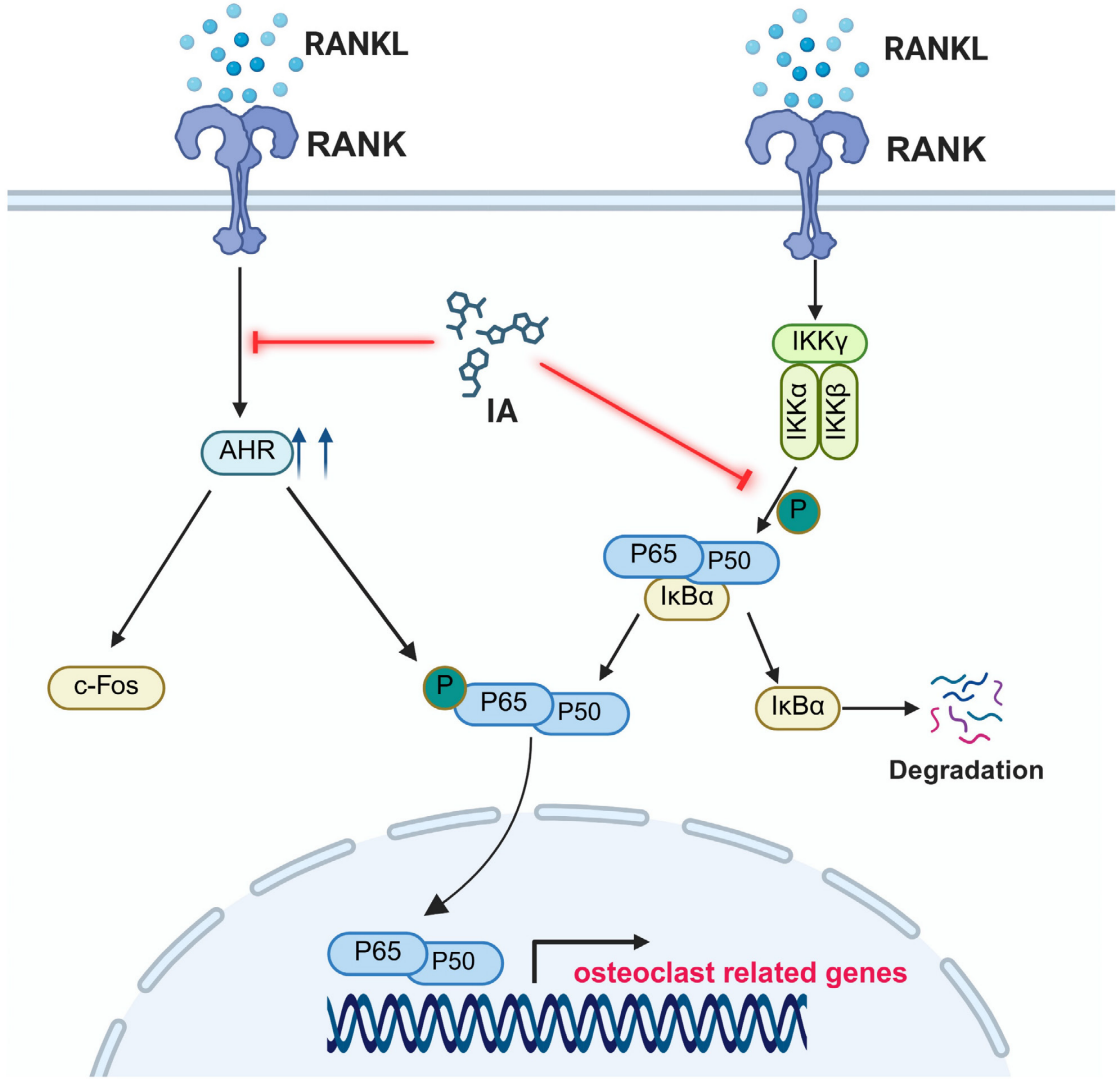
** A schematic diagram showing the working model**.

To further confirm the role of AhR, we generated AhR-overexpressing BMMs. Using these cells, AhR overexpression was shown to rescue the inhibitory effect of IA on RANKL-induced osteoclast differentiation, restoring both osteoclast formation and expression of osteoclast-specific markers. These findings indicated that IA inhibits osteoclastogenesis by suppressing AhR-mediated activation of the NF-κB signaling pathway. *In vivo*, 7 weeks of IA supplementation by oral gavage markedly alleviated OVX-induced bone loss. IA improved trabecular bone parameters, increased the bone formation marker PINP, and reduced the bone resorption marker CTX-I in OVX mice (Fig. 9).

Osteoclasts, the multinucleated giant cells responsible for bone resorption, are differentiated and activated through signaling cascades initiated by the binding of RANKL to its receptor RANK 38. RANKL induces c-Fos expression, which is required for subsequent NFATc1 induction and the full osteoclastogenic program 1. Although AhR has been identified as a negative regulator of osteoclastogenesis, different AhR ligands have been shown to exert a range of effects in osteoclast biology. These ligand-specific outcomes may reflect differences in dose, exposure duration, and the balance between classical and nonclassical AhR signaling pathways. However, the mechanisms by which AhR ligands regulate osteoclasts remain poorly understood. In the present study, IA induced downregulation of AhR in BMMs and disrupted the interaction between AhR and c-Fos, leading to reduced expression of downstream osteoclast-related genes. p65 (RelA) exists in the cytoplasm in a complex with IκBα. Upon activation of the NF-κB pathway, IKK phosphorylates IκBα, promoting its degradation and enabling p65 nuclear translocation to regulate target gene transcription 39. Notably, p65 participates in both AhR- and RANKL-mediated transcriptional programs 39. Our findings showed that IA inhibited RANKL-induced NF-κB activation, as evidenced by decreased phosphorylation of p65 and IκBα. These observations suggested that IA may limit RANKL-induced osteoclastogenesis by stimulating the AhR to compete with NF-κB in the nucleus.

AhR also plays an essential upstream role in regulating the expression of c-Fos. Izawa *et al.* 24 reported that overexpression of c-Fos rescued osteoclast function in AhR-knockout BMMs. Consistent with these observations, IA downregulated both c-Fos and AhR expression in our model, and we confirmed a physical interaction between both of these molecules. Taken together, these findings support a model in which IA inhibits osteoclast differentiation through the AhR-c-Fos axis and the downstream suppression of NF-κB (p65) signaling.

There is accumulating evidence that alterations in the gut microbiota and their metabolites are closely associated with decreased bone mass and the development of osteoporosis 40, 41. These microbial-derived factors are thought to modulate bone metabolism by influencing host metabolic, inflammatory, and immune status, thereby disrupting the balance between osteoclast and osteoblast activity. Supporting this suggestion, a recent study demonstrated that supplementation with IAA and IPA not only restored gut barrier integrity but also reduced systemic lipopolysaccharide (LPS) levels and M1 macrophage polarization in OVX mice 18. In our study, OVX mice showed a reduced abundance of *Lactobacillus* and lower serum IA levels. *Lactobacillus* species facilitate the conversion of tryptophan into IAA and IA 30, 31, 42, and numerous studies have shown that IA promotes intestinal epithelial barrier function and attenuates inflammatory responses 30, 31, 43, 44. Further, Wlodarska *et al.* demonstrated that IA exerted anti-inflammatory and antioxidative effects in LPS-activated human peripheral blood mononuclear cells (PBMCs) 45.

These findings suggest that IA supplementation exerts dual effects, with not only a direct inhibitory effect on osteoclast differentiation and resorption but also broader regulatory actions on intestinal barrier function and the systemic immune system. These combined effects may lead to increased bone mass. Future studies in our laboratory will explore the specific regulatory effects and mechanisms by which IA and IA-producing bacteria regulate the gut-bone axis.

Osteoblast-mediated bone formation is also important for maintaining the balance of bone metabolism. Therefore, we examined the effects of IA on osteogenic differentiation of BMSCs. *In vitro*, IA exerted neither promotive nor inhibitory effects on osteogenic differentiation of BMSCs. However, our *in vivo* animal experiments showed that IA supplementation not only inhibited bone resorption but also upregulated the osteogenic marker PINP in OVX mice. We speculate that the osteoporotic microenvironment is characterized by chronic inflammation and oxidative stress, which can impair osteoblast differentiation. Under such conditions, IA may exert indirect beneficial effects that enhance osteoblast function by mitigating inflammatory or oxidative stress signals, even though it does not directly stimulate osteogenesis under basal conditions *in vitro*. Further studies are required to investigate the mechanisms by which IA influences osteoblast activity in an inflammatory or oxidative microenvironment.

Targeting the gut microbiota and its metabolites represents a promising strategy for preventing and treating osteoporosis. Multiple interventions, including probiotics, prebiotics, dietary modifications, fecal microbiota transplantation (FMT), exercise, and specific nutritional supplements or pharmaceutical agents, are currently under investigation in preclinical and clinical studies for their potential to improve bone health 40.

In summary, our study showed that the gut-derived metabolite IA plays a key regulatory role in the development of postmenopausal osteoporosis in a mouse model. Serum IA concentration was strongly and positively correlated with bone mass. Mechanistically, IA significantly suppressed RANKL-induced osteoclast differentiation in BMMs through AhR-mediated inhibition of NF-κB signaling. We also confirmed that AhR functions as a positive regulator of osteoclastogenesis, as the inhibitory effect of IA on osteoclast formation were partially ameliorated by AhR overexpression, indicating that IA acts, at least in part, through the AhR-NF-κB axis (Fig. 9). Taken together, our findings provided mechanistic insights that may guide the development of novel therapeutic strategies for postmenopausal osteoporosis.

## Supplementary Material

Supplementary figures.

## Figures and Tables

**Table 1:** Sequences of primers for real-time quantitative PCR analysis

Gene name	Forward Primer (5'→3')	Reverse Primer (5'→3')
Nfatc1	CCGTTGCTTCCAGAAAATAACA	TGTGGGATGTGAACTCGGAA
c-Fos	CCAGTCAAGAGCATCAGCAA	AAGTAGTGCAGCCCGGAGTA
Mmp9	CAAAGACCTGAAAACCTCCAA	GGTACAAGTATGCCTCTGCCA
Cathepsin K	CTTCCAATACGTGCAGCAGA	TCTTCAGGGCTTTCTCGTTC
V-ATPase	GCCTCAGGGGAAGGCCAGATCG	GGCCACCTCTTCACTCCGGAA
Dc-stamp	AAAACCCTTGGGCTGTTCTT	AATCATGGACGACTCCTTGG
Ahr	GCTGAGGTGCCTGCTGGATAATTC	GCTCCGTCCTTCCCTTTCTTGTTC
Runx2	CACCTCGAATGGCAGCACGCTA	GCCGCCAAACAGACTCATCCA
Sp7	CCTAAGGGGCACAGCTCGTCT	TGCATGTCCCACCAAGGAGTAGG
Ocn	CAGTATGGCTTGAAGACCGC	GACATCCATACTTGCAGGGC
Opn	ATCTCACCATTCGGATGAGTCT	TGTAGGGACGATTGGAGTGAAA
GAPDH	GGCAAATTCAACGGCACAGTCAAG	TCGCTCCTGGAAGATGGTGATGG
338F_806R	ACTCCTACGGGAGGCAGCAG	GGACTACHVGGGTWTCTAAT

## Abbreviations

SCFA: Short-Chain Fatty Acids
TMAO: Trimethylamine-N-oxide
OIM: Osteogenic Induction Medium
AHR: Aryl Hydrocarbon Receptor
HSP: Heat Shock Protein
ESR: Estrogen receptor
Nrf2: Nuclear factor erythroid 2-related factor 2
NF-κB: Nuclear Factor kappa-B
BMM: Bone Marrow Macrophage
BMSC: Bone marrow mesenchymal stem cells
NFATc1: nuclear factor of activated T-cells, cytoplasmic 1
CTSK: Cathepsin K
MMP9: Matrix metallopeptidase 9
BV/TV: Bone volume fraction
Tb.N: Trabecular number
Tb.Th: Trabecular thickness
Tb.Sp: Trabecular separation
FBS: Fetal Bovine Serum
BSA: Bovine Serum Albumin
PFA: Paraformaldehyde
TRAP: Tartrate-Resistant Acid Phosphatase
CTX-1: Type I Collagen C-Terminal Telopeptide
P1NP: propeptide of type I procollagen
ALP: Alkaline Phosphatase
AST: Aspartate Aminotransferase
