# Supplementary material for: Gut Metabolite Indoleacrylic Acid Suppresses Osteoclast Formation by AHR mediated NF-κB Signaling Pathway
Source: Int J Biol Sci. 2026 Jan 1;22(2):951–69. doi: 10.7150/ijbs.124766 (PMC12781171; doi:10.7150/ijbs.124766)
Supplement: Supplementary file 1 — Supplementary figures. [file ijbsv22p0951s1.pdf]

Gut Metabolite Indoleacrylic Acid Suppresses Osteoclast Formation by AHR mediated NF-κB Signaling Pathway

Jinwu Bai[1,2,#], Gao Si[1,2,#], Ruideng Wang[3,#], Shilong Su[1,2], Jixing Fan[1,2], Xi He[4], Yang Lv[1,2,*], Shan Gao[1,2,*], Fang Zhou[1,2,*]

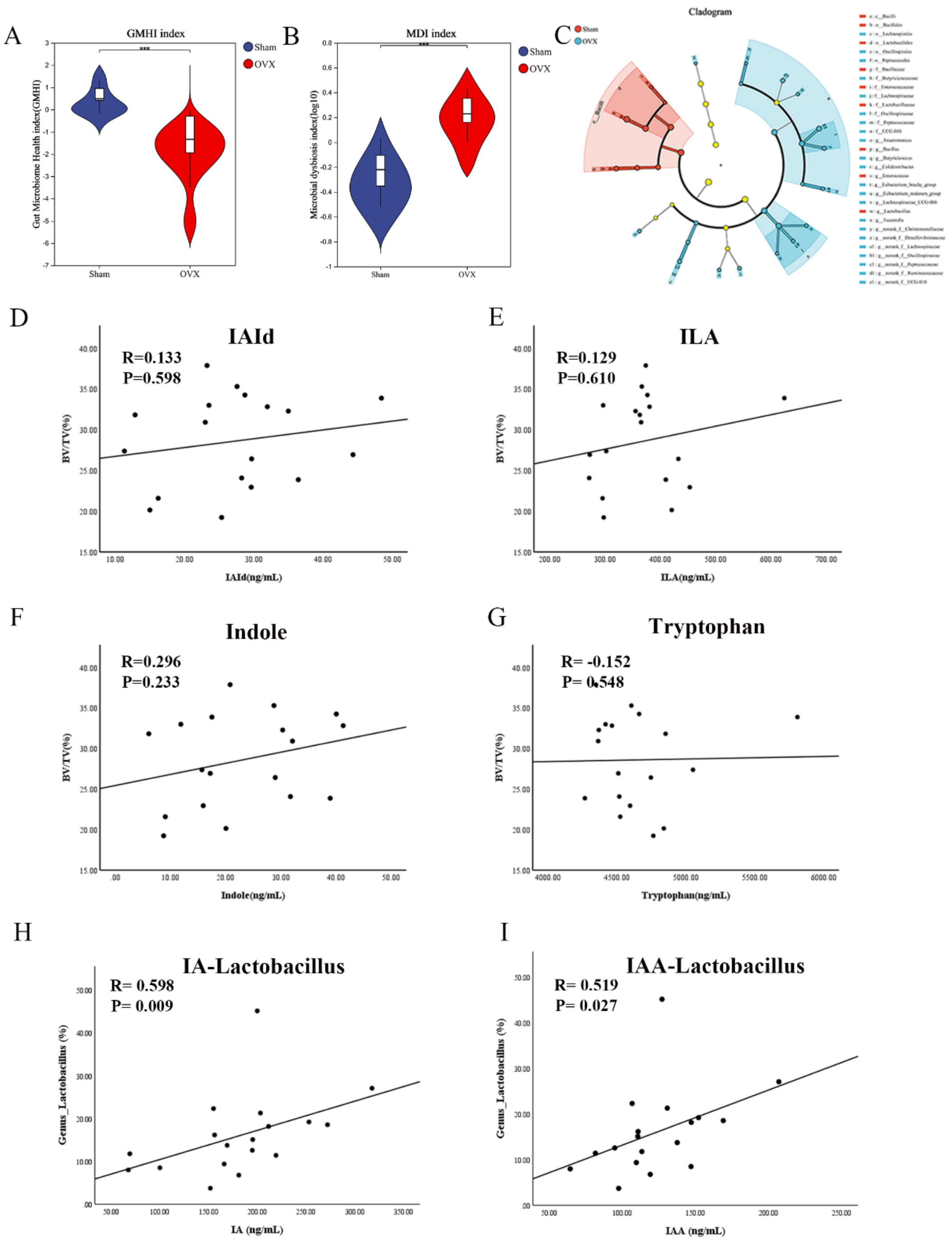

Fig. S1 Altered gut microbiota and correlation of tryptophan metabolites. (A, B) The GMHI index and MDI index of Sham and OVX group, n=9. (C) Analysis of the relative abundance of taxa among different groups using a LEfSe cladogram. LDA effect size cladograms showing the taxa most differentially associated with Sham (green) or OVX mice (orange) (Wilcoxon rank-sum test). Circle sizes in the cladogram plot are proportional to bacterial abundance. The circles represent, going from the inner to outer circle: genus, class, order, family, and phylum. LDA≥2.0 and p≤0.05 (Wilcoxon rank-sum test) was regarded as significance. (D, E, F, G) Spearman correlation analysis between the BV/TV and microbial tryptophan metabolites (IAId, ILA, indole and tryptophan). (H, I) Spearman correlation analysis between the IA, IAA and the abundance of Lactobacillus.

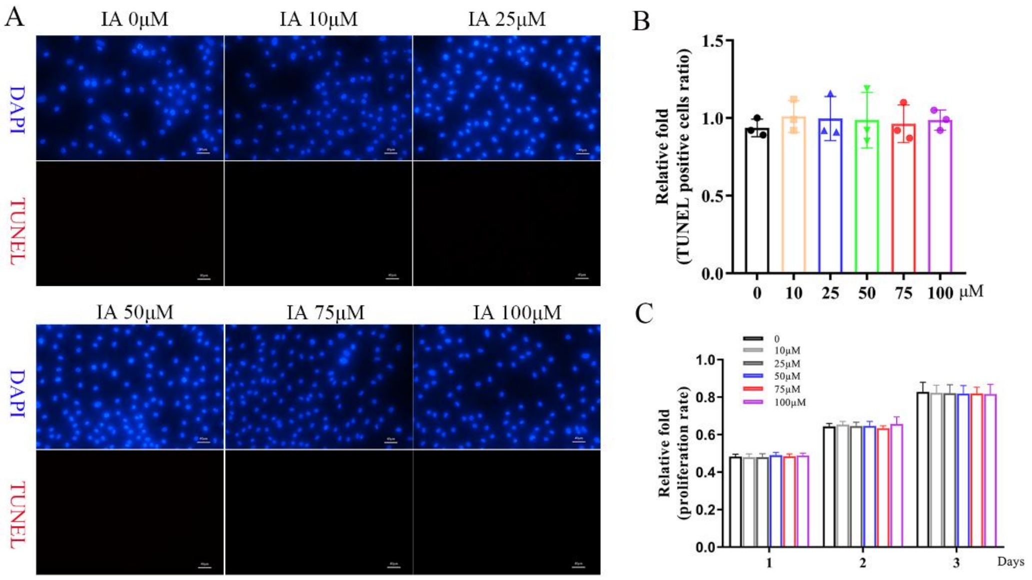

Fig. S2 IA had no cytotoxicity on cell proliferation and apoptosis. (A) The apoptosis of BMMs was observed by TUNEL staining at 4 days. (B) The quantitative analysis of TUNEL staining. (C) CCK-8 assay was utilized to assess the cell viability of BMMs. Data are shown as mean±SD, n=3, *p<0.05 **p<0.01, ns: no significant differences.

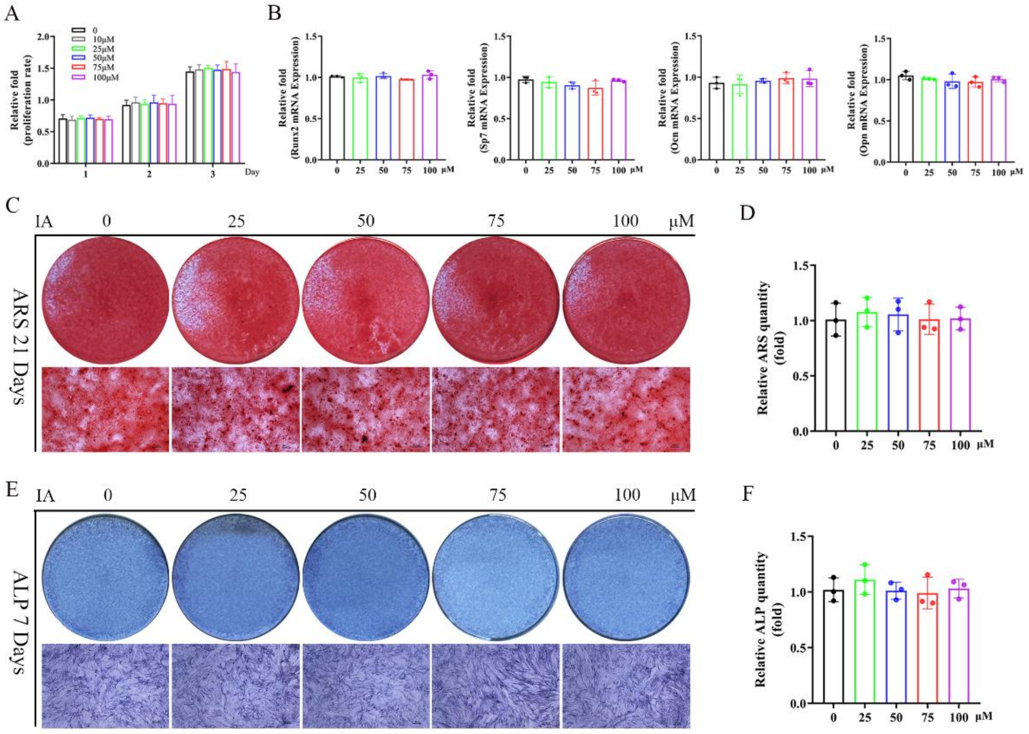

Fig. S3 Fig. S3 IA did not directly affect osteoblastic differentiation. (A) CCK-8 assay was utilized to assess the cell viability of BMSCs. (B) The mRNA levels of key osteogenic related genes were evaluated by qRT-PCR analysis after 4 days OIM. (C) The osteoblastic commitment of BMSCs after Garcinol administration was determined by ALP staining. Scale bar=100μm. (D) The quantification of ALP activity. (E) The mineralized depositions of BMSCs were measured by ARS staining. Scale bar=100μm. (F) The quantification of ARS staining. Data are shown as mean±SD, n=3, *p<0.05 **p<0.01, ns: no significant differences.

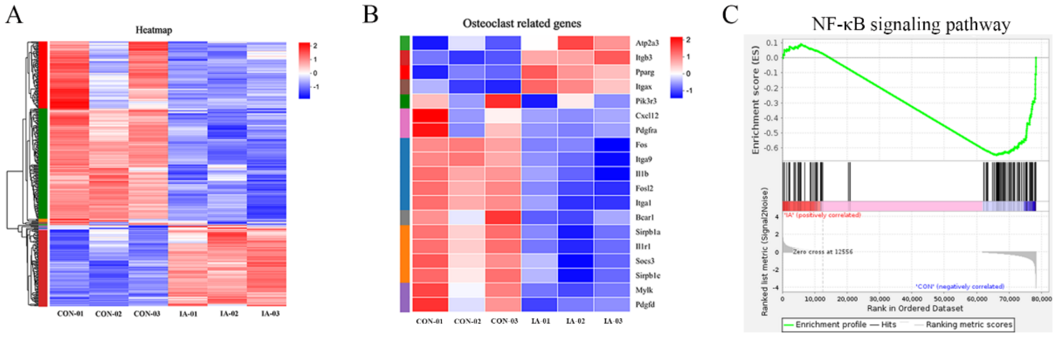

Fig. S4 IA suppresses RANKL-induced NF-κB signaling pathway. (A) Heat maps showing differentially expressed genes between the control and IA treatment groups. (B) Heat maps showing osteoclast related genes with IA treatment between two groups. (C) GSEA analysis confirmed the inhibition of the NF-κB signaling pathways by IA.

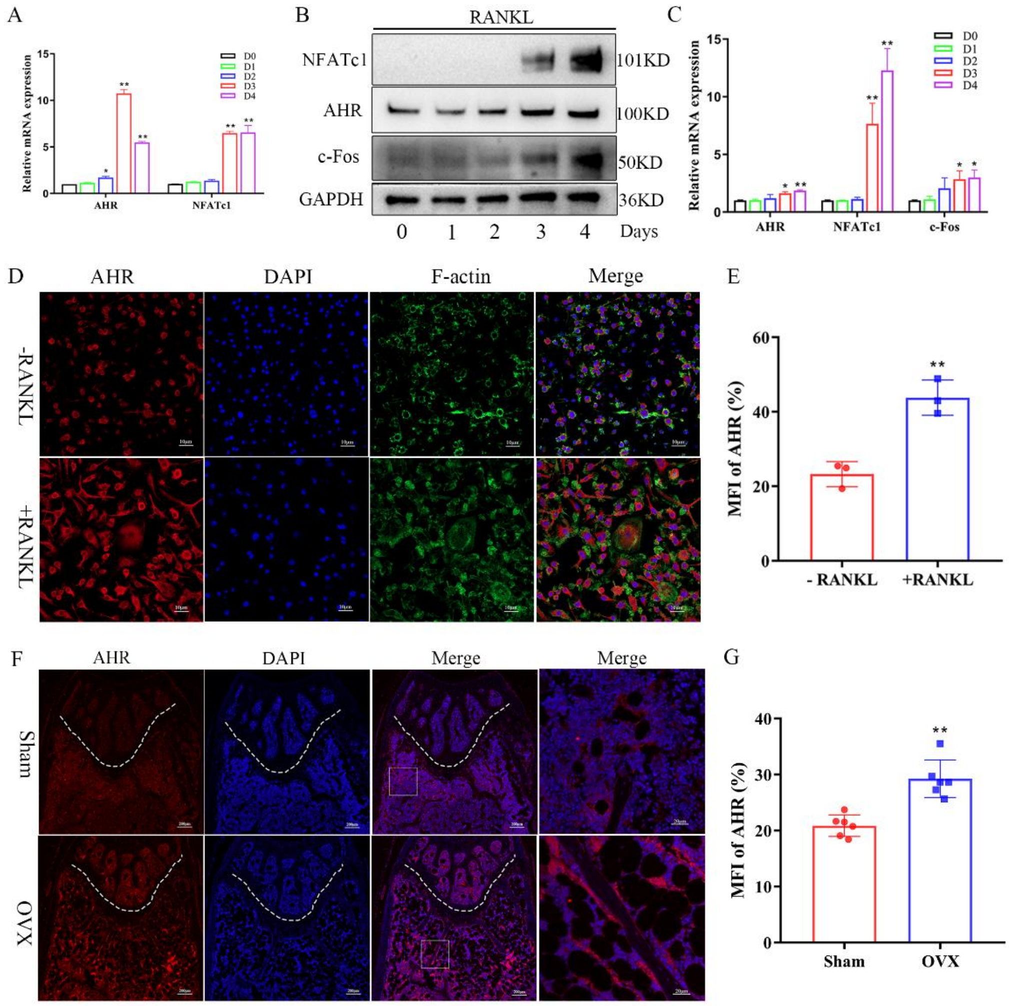

Fig. S5 AHR is upregulated during osteoclastogenesis. (A) mRNA expression of Ahr and Nfatc1 in BMMs during the RANKL induced osteoclast differentiation. (B) Protein expression of Ahr, Nfatc1 and c-Fos in BMMs during the RANKL induced osteoclast differentiation. (C) Quantification of protein of western blot. (D, E) AHR (red) and F-actin (green) immunofluorescence staining and quantification of BMMs with or without osteoclastogenesis. Scale bar, 10μm, (F, G) AHR (red) immunofluorescence staining and quantification of femora sections from Sham and OVX mice. n=6, Scale bar, 200μm, 20μm. Data are presented as means ± SD of 3 independent experiments; *$p<0.05$ **$p<0.01$, ns: no significant differences.

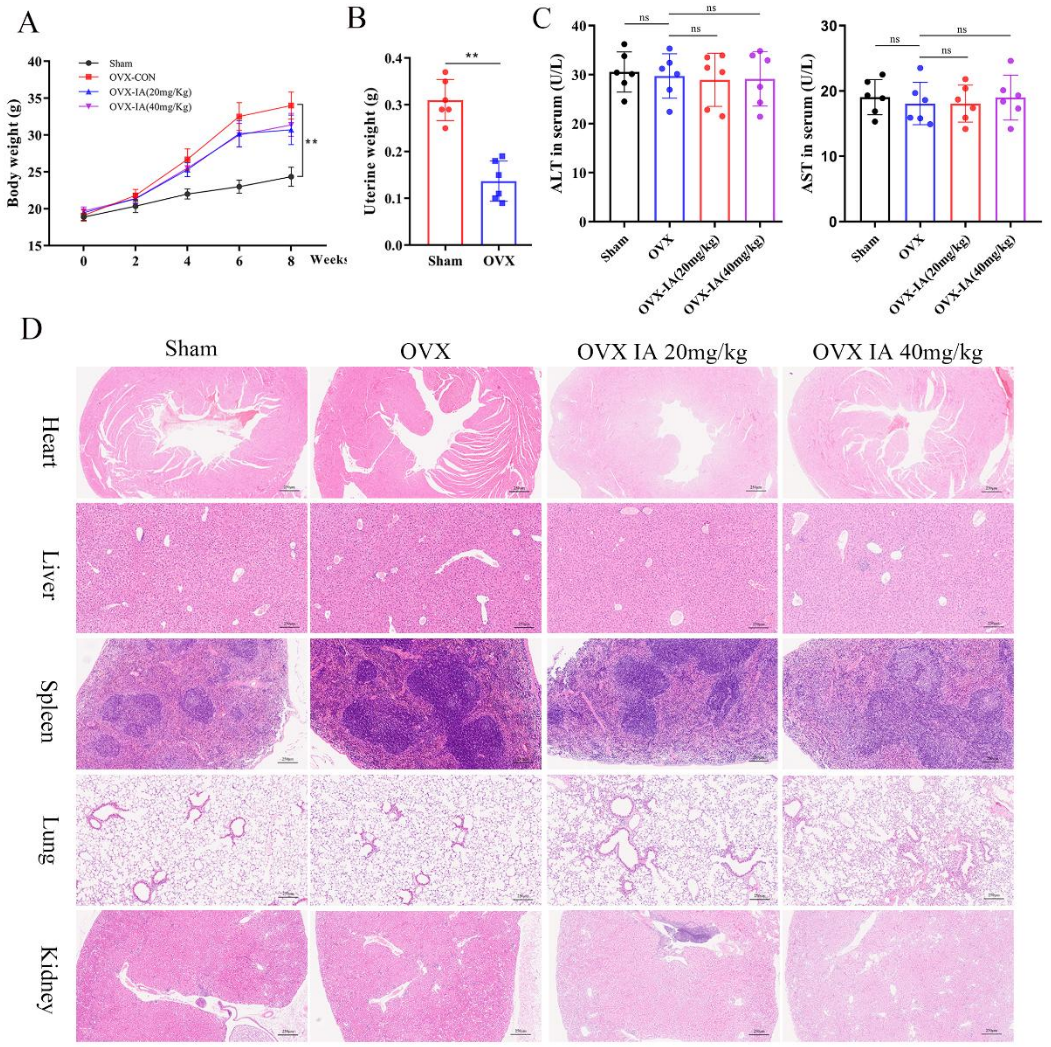

Fig. S6 IA supplementation has no obvious toxicity of whole body. (A) The whole-body weight of mice during different stage. (B) The uterine weight of mice at the time of sacrifice. (C) The levels of serum ALT and AST among the groups. (D) Representative pictures in H&E staining of Heart, liver, spleen, lung and kidney. scale bar, 100 µm; Data are presented as means ± SD. (n = 6) *p<0.05 **p<0.01, ns: no significant differences.
